# Bis(diethyl­amido-κ*N*)(diethyl­amine-κ*N*)bis­(2,6-diisopropyl­phenyl­amido-κ*N*)zirconium(IV)

**DOI:** 10.1107/S160053681205115X

**Published:** 2013-01-04

**Authors:** Mateusz Zauliczny, Rafał Grubba, Łukasz Ponikiewski, Jerzy Pikies

**Affiliations:** a Chemical Faculty, Gdansk University of Technology, Gabriela Narutowicza Street 11/12, 80-233 Gdansk, Poland

## Abstract

In the title compound, [Zr(C_12_H_18_N)_2_(C_4_H_10_N)_2_(C_4_H_11_N)] or [Zr(HNC_6_H_3_^i^Pr_2_)_2_(NEt_2_)_2_(HNEt_2_)], which was obtained by the reaction of Zr(NEt)_4_ with ^i^Pr_2_C_6_H_3_NH_2_, the Zr^IV^ atom is in a trigonal–bipiramidal geometry in which the N atoms from two ^i^Pr_2_C_6_H_3_NH and one NEt_2_ ligand occupy the equatorial positions, and the N atoms of an NEt_2_ and an Et_2_NH ligand occupy the apical positions. An intra­molecular N—H⋯N contact occurs. There are two independent molecules in the asymmetric unit.

## Related literature

For related zirconium(IV) structures, see: Profilet *et al.* (1990 ▶); Blake *et al.* (1997 ▶); Porter & Danopoulos (2004 ▶); Ghesner *et al.* (2006 ▶). For related syntheses, see: Kempe (2000 ▶).
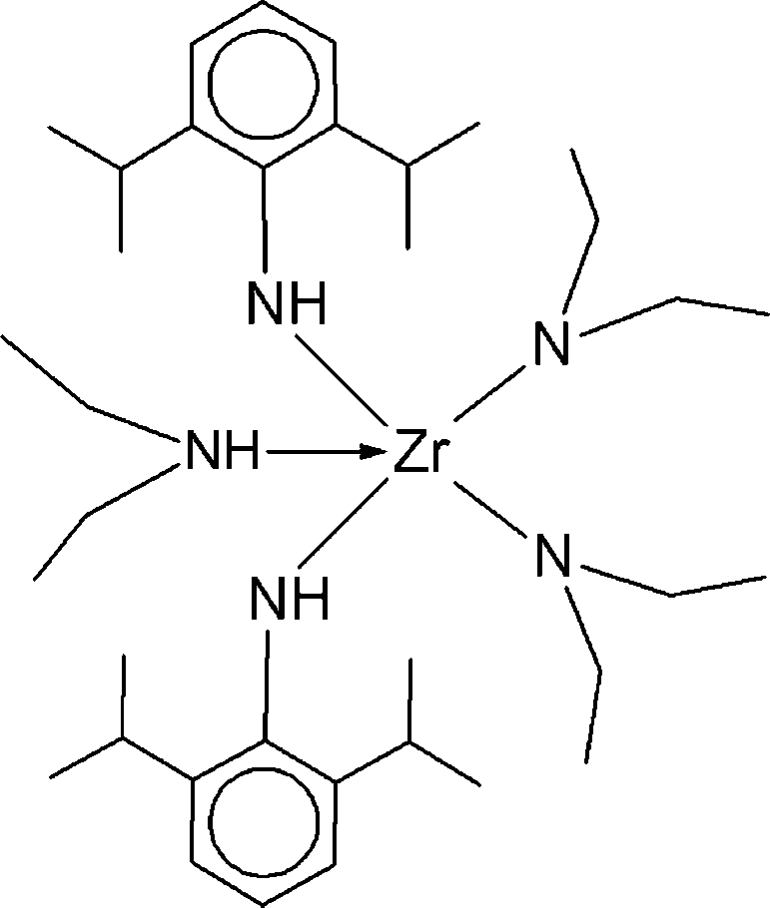


## Experimental

### 

#### Crystal data

[Zr(C_12_H_18_N)_2_(C_4_H_10_N)_2_(C_4_H_11_N)]
*M* *_r_* = 661.17
Triclinic, 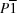

*a* = 11.2079 (3) Å
*b* = 13.1612 (5) Å
*c* = 14.3443 (6) Å
α = 86.578 (3)°
β = 70.484 (3)°
γ = 71.232 (3)°
*V* = 1885.61 (12) Å^3^
*Z* = 2
Mo *K*α radiation
μ = 0.32 mm^−1^
*T* = 293 K
0.58 × 0.39 × 0.34 mm

#### Data collection

Agilent Xcalibur (Sapphire2 diffractometer
Absorption correction: analytical (*CrysAlis PRO*; Agilent, 2010 ▶) *T*_min_ = 0.889, *T*_max_ = 0.928
11493 measured reflections
7412 independent reflections
5867 reflections with *I* > 2σ(*I*)
*R*_int_ = 0.035

#### Refinement

*R*[*F* ^2^ > 2σ(*F* ^2^)] = 0.073
*wR*(*F* ^2^) = 0.188
*S* = 1.12
7412 reflections
402 parameters
1 restraint
H atoms treated by a mixture of independent and constrained refinement
Δρ_max_ = 1.82 e Å^−3^
Δρ_min_ = −2.13 e Å^−3^

### 

Data collection: *CrysAlis PRO* (Agilent, 2010 ▶); cell refinement: *CrysAlis PRO*; data reduction: *CrysAlis PRO*; program(s) used to solve structure: *SUPERFLIP* (Palatinus & Chapuis, 2007 ▶); program(s) used to refine structure: *SHELXL97* (Sheldrick, 2008 ▶); molecular graphics: *ORTEP-3 for Windows* (Farrugia, 2012 ▶); software used to prepare material for publication: *WinGX* (Farrugia, 2012 ▶).

## Supplementary Material

Crystal structure: contains datablock(s) global, I. DOI: 10.1107/S160053681205115X/ng5310sup1.cif

Structure factors: contains datablock(s) I. DOI: 10.1107/S160053681205115X/ng5310Isup2.hkl

Additional supplementary materials:  crystallographic information; 3D view; checkCIF report

## Figures and Tables

**Table 1:** Hydrogen-bond geometry (Å, °)

*D*—H⋯*A*	*D*—H	H⋯*A*	*D*⋯*A*	*D*—H⋯*A*
N3—H3*A*⋯N1	0.84 (5)	2.56 (5)	2.983 (5)	112 (4)

**Fig. 1.**
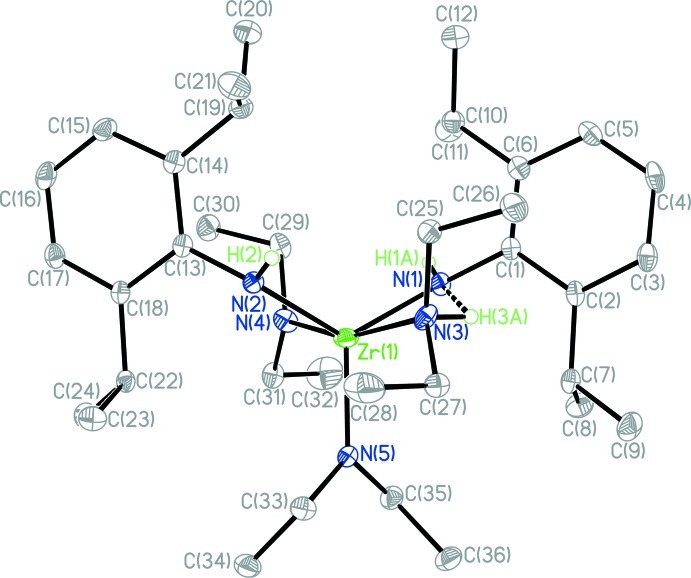
The molecular structure of the title molecule with the atom-numbering. Displacement ellipsoids are drawn at the 30% probability level H atoms connected to C have been omitted.

**Fig. 2.**
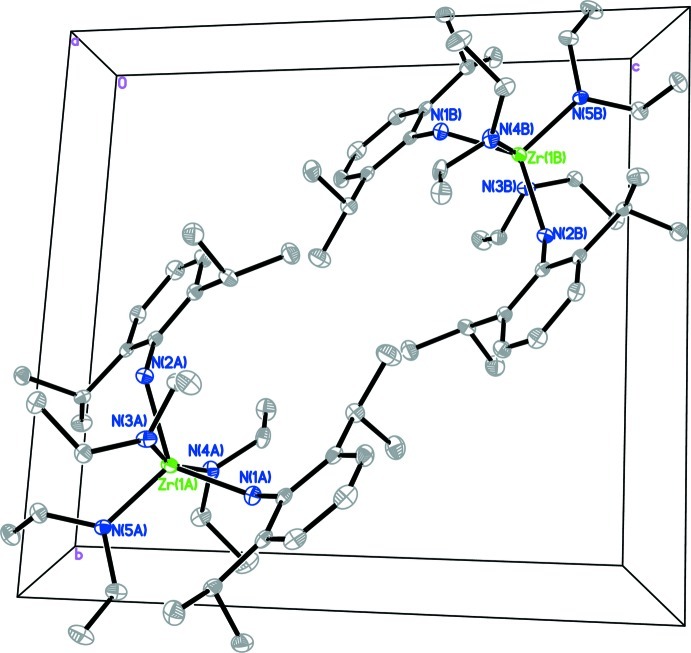
A view of the packing of the title compound alond the a axix.

## supplementary crystallographic information

### Comment

Complex (I) was synthesized in the course of our studies on amido complexes of zirconium (Kempe, 2000). The compound was obtained in the reaction Zr(NEt)_4_ with ^i^Pr_2_C_6_H_3_NH_2_ (molar ratio 1:2). Complex (I) contains four amido ligands (two NEt_2_ and two ^i^Pr_2_C_6_H_3_NH) and one amino ligand (HNEt_2_). Analyzing bond lengths in title compound it is easily spotted that bond Zr—N3 is much longer than other bonds. It is caused by the fact, that ligand containing N1 is amine ligand. Difference between length of bonds Zr—N between NEt_2_ and HNEt_2_ is about 0.25 Å. Distances between N atoms of ^i^Pr_2_C_6_H_3_NH and Zr are both about 2.12 Å and very similar to related Zr(IV) amido complexes (Profilet *et al.*, 1990; Blake *et al.*, 1997). In case of two NEt_2_ ligands distances Zr—N differ by about 0.12 Å but both are in the range typical for zirconium complexes with diethylamido ligands (Porter *et al.,* 2004; Ghesner *et al.*, 2006). Comparing angles between N1—Zr1—N2, N2—Zr1—N5 and N5—Zr1—N1 [table 1] it can be seen that they are roughly 120°, in addition the angle between N3—Zr1—N4 [table 1] indicates that molecular geometry is close to trigonal bipyramidal. Admittedly in perfect trigonal bipyramid first three angles would be equal to 120° and N3—Zr1—N4 would be equal to 180°, but actual angles are so close to said values that it is safe to say they resemble trigonal bipyramid.

The crystal packing diagram shows, that the compound crystallizes with two molecules in the unit cell in the triclinic space group. The crystal packing of the title compound is presented in Fig.2.

### Experimental

To a 100 ml Schlenk flask, equipped with a magnetic stirrer, charged with a solution of 4,8 g (4,44 ml) Zr(NEt_2_)_4_ in 30 ml of pentane, 5 g (4,76 ml) of 2,6-diisopropylaniline in 10 ml of pentane was added dropwise. The reaction was carried on in a room temperature in an argon atmosphere. Solution was left on a magnetic stirrer. Over a week of stirring, the mixture changed colour from yellow to brown. The solvent was removed under vacuum. After evaporating most of the solvent, dark solid was obtained which after keeping it longer under vacuum got darker and became oil. Residue was dissolved in 8 ml of pentane, and recrystallized at 4°C to obtain about 2 g of colorless X-ray-quality crystals. The total yield was 24%. Elemental analysis, found %: C 65.73, H 9.72, N 10.46; calc. % for C_30_H_52_N_5_Zr: C 65.40, H 10.21, N 10.59.

### Refinement

The C—H H atoms were positioned with idealized geometry and were refined isotropically with *U*_iso_(H) = 1.2*U*_eq_(C) for aromatic, methylene and methine H atoms (1.5 for methyl H atoms) using a riding model with C—H = 0.93 Å (aromatic H atoms), 0.96 Å (methyl H atoms), 0.97 Å (methylene H atoms) and 0.98 Å (methine H atoms).The amine hydrogen atoms were located in the difference Fourier map and refined using a ridnig model with *U*_iso_(H) = 1.2*U*_eq_(N). The hydrogen atom H1A is located in the difference map and restrained, N1-H1A = 0.89 Å with *U*_iso_(H) = 1.2*U*_eq_(N). The highest residual electron density peaks are located within 1 Å from atom Zr1 and the deepest hole is located 0.78 Å from Zr1.

### Crystal data

**Table:**

[Zr(C_12_H_18_N)_2_(C_4_H_10_N)_2_(C_4_H_11_N)]	*Z* = 2
*M**_r_* = 661.17	*F*(000) = 716
Triclinic, *P*1	*D*_x_ = 1.164 Mg m^−^^3^
Hall symbol: -P 1	Mo *K*α radiation, λ = 0.71073 Å
*a* = 11.2079 (3) Å	Cell parameters from 6954 reflections
*b* = 13.1612 (5) Å	θ = 2.9–28.4°
*c* = 14.3443 (6) Å	µ = 0.32 mm^−^^1^
α = 86.578 (3)°	*T* = 293 K
β = 70.484 (3)°	Block, colourless
γ = 71.232 (3)°	0.58 × 0.39 × 0.34 mm
*V* = 1885.61 (12) Å^3^	

### Data collection

**Table:**

Agilent Xcalibur (Sapphire2 diffractometer	7412 independent reflections
Graphite monochromator	5867 reflections with *I* > 2σ(*I*)
Detector resolution: 8.1883 pixels mm^-1^	*R*_int_ = 0.035
ω scans	θ_max_ = 26°, θ_min_ = 2.9°
Absorption correction: analytical (*CrysAlis PRO*; Agilent, 2010)	*h* = −13→8
*T*_min_ = 0.889, *T*_max_ = 0.928	*k* = −16→12
11493 measured reflections	*l* = −17→12

### Refinement

**Table:**

Refinement on *F*^2^	Primary atom site location: structure-invariant direct methods
Least-squares matrix: full	Secondary atom site location: difference Fourier map
*R*[*F*^2^ > 2σ(*F*^2^)] = 0.073	Hydrogen site location: inferred from neighbouring sites
*wR*(*F*^2^) = 0.188	H atoms treated by a mixture of independent and constrained refinement
*S* = 1.12	*w* = 1/[σ^2^(*F*_o_^2^) + (0.0694*P*)^2^ + 9.6553*P*] where *P* = (*F*_o_^2^ + 2*F*_c_^2^)/3
7412 reflections	(Δ/σ)_max_ < 0.001
402 parameters	Δρ_max_ = 1.82 e Å^−^^3^
1 restraint	Δρ_min_ = −2.13 e Å^−^^3^

### Special details

**Table:**

Geometry. All e.s.d.'s (except the e.s.d. in the dihedral angle between two l.s. planes) are estimated using the full covariance matrix. The cell e.s.d.'s are taken into account individually in the estimation of e.s.d.'s in distances, angles and torsion angles; correlations between e.s.d.'s in cell parameters are only used when they are defined by crystal symmetry. An approximate (isotropic) treatment of cell e.s.d.'s is used for estimating e.s.d.'s involving l.s. planes.
Refinement. Refinement of *F*^2^ against ALL reflections. The weighted *R*-factor *wR* and goodness of fit *S* are based on *F*^2^, conventional *R*-factors *R* are based on *F*, with *F* set to zero for negative *F*^2^. The threshold expression of *F*^2^ > σ(*F*^2^) is used only for calculating *R*-factors(gt) *etc*. and is not relevant to the choice of reflections for refinement. *R*-factors based on *F*^2^ are statistically about twice as large as those based on *F*, and *R*- factors based on ALL data will be even larger.

### Fractional atomic coordinates and isotropic or equivalent isotropic displacement parameters (Å^2^)

**Table:**

	*x*	*y*	*z*	*U*_iso_*/*U*_eq_	
Zr1	0.73585 (6)	0.79022 (4)	0.21365 (4)	0.03188 (17)	
N1	0.7260 (3)	0.8397 (3)	0.3554 (3)	0.0216 (8)	
H1A	0.650 (3)	0.838 (4)	0.401 (3)	0.026*	
N2	0.7770 (4)	0.6310 (3)	0.1615 (3)	0.0199 (8)	
H2	0.856 (5)	0.591 (4)	0.160 (4)	0.024*	
N3	0.9717 (4)	0.7334 (3)	0.1861 (3)	0.0244 (8)	
H3A	0.963 (5)	0.779 (4)	0.229 (4)	0.029*	
N4	0.5246 (4)	0.8112 (3)	0.2736 (3)	0.0277 (9)	
N5	0.7378 (3)	0.9016 (3)	0.1088 (3)	0.0197 (7)	
C1	0.8144 (4)	0.8339 (3)	0.4082 (3)	0.0196 (9)	
C2	0.8987 (4)	0.8990 (4)	0.3821 (3)	0.0207 (9)	
C3	0.9889 (5)	0.8903 (4)	0.4321 (4)	0.0285 (10)	
H3	1.0441	0.9331	0.4152	0.034*	
C4	0.9975 (5)	0.8195 (4)	0.5060 (4)	0.0320 (11)	
H4	1.0588	0.8141	0.5381	0.038*	
C5	0.9145 (5)	0.7562 (4)	0.5325 (4)	0.0306 (11)	
H5	0.9202	0.7093	0.5831	0.037*	
C6	0.8225 (4)	0.7616 (4)	0.4847 (3)	0.0248 (10)	
C7	0.8854 (4)	0.9830 (4)	0.3060 (3)	0.0242 (9)	
H7	0.8631	0.9538	0.2552	0.029*	
C8	0.7667 (5)	1.0843 (4)	0.3554 (4)	0.0349 (12)	
H8A	0.6871	1.0649	0.3849	0.052*	
H8B	0.7539	1.1357	0.3064	0.052*	
H8C	0.7855	1.1153	0.4057	0.052*	
C9	1.0102 (5)	1.0135 (5)	0.2538 (4)	0.0367 (12)	
H9A	1.0316	1.0469	0.301	0.055*	
H9B	0.9947	1.0627	0.2037	0.055*	
H9C	1.0833	0.95	0.2236	0.055*	
C10	0.7317 (5)	0.6931 (4)	0.5149 (4)	0.0277 (10)	
H10	0.7206	0.672	0.4547	0.033*	
C11	0.5927 (5)	0.7584 (5)	0.5843 (4)	0.0404 (13)	
H11A	0.5981	0.7717	0.6475	0.061*	
H11B	0.5318	0.7186	0.5925	0.061*	
H11C	0.5612	0.8256	0.5561	0.061*	
C12	0.7852 (6)	0.5903 (5)	0.5637 (5)	0.0453 (14)	
H12A	0.871	0.5482	0.52	0.068*	
H12B	0.7244	0.5496	0.5773	0.068*	
H12C	0.7937	0.6083	0.6246	0.068*	
C13	0.6972 (4)	0.5653 (3)	0.1692 (3)	0.0192 (9)	
C14	0.7022 (4)	0.4784 (4)	0.2323 (3)	0.0226 (9)	
C15	0.6217 (5)	0.4149 (4)	0.2379 (3)	0.0282 (10)	
H15	0.6266	0.357	0.2784	0.034*	
C16	0.5353 (5)	0.4361 (4)	0.1847 (4)	0.0306 (11)	
H16	0.4813	0.3937	0.1899	0.037*	
C17	0.5298 (5)	0.5218 (4)	0.1233 (3)	0.0261 (10)	
H17	0.4718	0.5357	0.087	0.031*	
C18	0.6073 (4)	0.5871 (3)	0.1142 (3)	0.0193 (9)	
C19	0.7952 (5)	0.4533 (4)	0.2924 (3)	0.0267 (10)	
H19	0.8076	0.5209	0.3049	0.032*	
C20	0.7410 (6)	0.4094 (4)	0.3930 (4)	0.0375 (12)	
H20A	0.6539	0.4573	0.4286	0.056*	
H20B	0.8	0.4035	0.43	0.056*	
H20C	0.7351	0.3398	0.3836	0.056*	
C21	0.9322 (5)	0.3769 (5)	0.2345 (4)	0.0390 (12)	
H21A	0.9246	0.3085	0.2232	0.058*	
H21B	0.9916	0.3682	0.2716	0.058*	
H21C	0.9668	0.406	0.172	0.058*	
C22	0.6044 (4)	0.6763 (3)	0.0418 (3)	0.0210 (9)	
H22	0.6282	0.7316	0.0675	0.025*	
C23	0.4647 (5)	0.7303 (4)	0.0324 (4)	0.0306 (11)	
H23A	0.4414	0.6797	0.0022	0.046*	
H23B	0.4661	0.7911	−0.008	0.046*	
H23C	0.3999	0.7538	0.0969	0.046*	
C24	0.7096 (5)	0.6349 (4)	−0.0588 (3)	0.0294 (10)	
H24A	0.7965	0.6084	−0.0518	0.044*	
H24B	0.7071	0.6924	−0.1033	0.044*	
H24C	0.6918	0.578	−0.0848	0.044*	
C25	1.0286 (4)	0.6345 (4)	0.2313 (3)	0.0251 (10)	
H25A	1.0531	0.5737	0.1859	0.03*	
H25B	0.9602	0.6261	0.2911	0.03*	
C26	1.1508 (5)	0.6316 (5)	0.2573 (4)	0.0380 (12)	
H26A	1.2205	0.6373	0.1982	0.057*	
H26B	1.1814	0.5652	0.2868	0.057*	
H26C	1.1274	0.6907	0.3033	0.057*	
C27	1.0646 (4)	0.7550 (4)	0.0928 (3)	0.0239 (9)	
H27A	1.1431	0.7593	0.1047	0.029*	
H27B	1.0217	0.8239	0.0707	0.029*	
C28	1.1072 (7)	0.6691 (5)	0.0121 (4)	0.0509 (16)	
H28A	1.1663	0.6043	0.027	0.076*	
H28B	1.1527	0.6931	−0.0502	0.076*	
H28C	1.0298	0.6555	0.0081	0.076*	
C29	0.4813 (5)	0.7500 (4)	0.3598 (3)	0.0282 (10)	
H29A	0.4477	0.7966	0.419	0.034*	
H29B	0.5585	0.6917	0.3636	0.034*	
C30	0.3737 (5)	0.7030 (5)	0.3591 (4)	0.0366 (12)	
H30A	0.2926	0.7603	0.364	0.055*	
H30B	0.3577	0.6583	0.4143	0.055*	
H30C	0.403	0.6608	0.2985	0.055*	
C31	0.4177 (4)	0.9023 (4)	0.2580 (4)	0.0292 (10)	
H31A	0.3505	0.8756	0.2497	0.035*	
H31B	0.4546	0.9342	0.1969	0.035*	
C32	0.3507 (8)	0.9887 (5)	0.3406 (5)	0.067 (2)	
H32A	0.3025	0.9608	0.3992	0.101*	
H32B	0.2898	1.0489	0.3214	0.101*	
H32C	0.4173	1.0112	0.3536	0.101*	
C33	0.7937 (4)	0.8757 (4)	0.0015 (3)	0.0268 (10)	
H33A	0.8264	0.7982	−0.0097	0.032*	
H33B	0.8697	0.9015	−0.0258	0.032*	
C34	0.6955 (5)	0.9233 (4)	−0.0547 (4)	0.0344 (12)	
H34A	0.6159	0.9041	−0.0243	0.052*	
H34B	0.736	0.8955	−0.1223	0.052*	
H34C	0.6729	1.0002	−0.0527	0.052*	
C35	0.6795 (5)	1.0173 (4)	0.1362 (4)	0.0297 (10)	
H35A	0.6508	1.027	0.2077	0.036*	
H35B	0.6006	1.0457	0.1166	0.036*	
C36	0.7729 (6)	1.0832 (4)	0.0903 (4)	0.0405 (13)	
H36A	0.8553	1.0516	0.1035	0.061*	
H36B	0.7313	1.1555	0.1186	0.061*	
H36C	0.7906	1.0839	0.0201	0.061*	

### Atomic displacement parameters (Å^2^)

**Table:**

	*U*^11^	*U*^22^	*U*^33^	*U*^12^	*U*^13^	*U*^23^
Zr1	0.0557 (4)	0.0191 (2)	0.0234 (3)	−0.0121 (2)	−0.0168 (2)	0.00391 (17)
N1	0.0115 (17)	0.028 (2)	0.0236 (19)	−0.0066 (15)	−0.0036 (14)	−0.0005 (16)
N2	0.0132 (18)	0.0206 (19)	0.0262 (19)	−0.0045 (15)	−0.0079 (15)	0.0025 (15)
N3	0.0156 (19)	0.0203 (19)	0.035 (2)	−0.0068 (15)	−0.0042 (16)	−0.0001 (16)
N4	0.0171 (19)	0.035 (2)	0.025 (2)	−0.0036 (17)	−0.0046 (15)	0.0012 (17)
N5	0.0151 (18)	0.0180 (18)	0.0237 (19)	−0.0038 (14)	−0.0047 (14)	−0.0006 (14)
C1	0.013 (2)	0.021 (2)	0.020 (2)	−0.0019 (17)	−0.0023 (16)	−0.0057 (17)
C2	0.011 (2)	0.025 (2)	0.022 (2)	−0.0028 (17)	−0.0021 (16)	−0.0082 (17)
C3	0.018 (2)	0.031 (3)	0.036 (3)	−0.0060 (19)	−0.0078 (19)	−0.007 (2)
C4	0.018 (2)	0.041 (3)	0.040 (3)	−0.004 (2)	−0.015 (2)	−0.008 (2)
C5	0.033 (3)	0.035 (3)	0.026 (2)	−0.007 (2)	−0.015 (2)	0.001 (2)
C6	0.023 (2)	0.024 (2)	0.022 (2)	−0.0027 (19)	−0.0049 (18)	−0.0056 (18)
C7	0.023 (2)	0.024 (2)	0.026 (2)	−0.0083 (19)	−0.0070 (18)	−0.0045 (18)
C8	0.035 (3)	0.025 (3)	0.038 (3)	−0.005 (2)	−0.008 (2)	0.000 (2)
C9	0.030 (3)	0.048 (3)	0.035 (3)	−0.023 (2)	−0.004 (2)	0.003 (2)
C10	0.025 (2)	0.029 (3)	0.029 (2)	−0.009 (2)	−0.0093 (19)	0.003 (2)
C11	0.035 (3)	0.048 (3)	0.033 (3)	−0.018 (3)	0.000 (2)	0.004 (2)
C12	0.059 (4)	0.036 (3)	0.053 (4)	−0.019 (3)	−0.031 (3)	0.013 (3)
C13	0.012 (2)	0.022 (2)	0.020 (2)	−0.0049 (17)	−0.0014 (16)	−0.0032 (17)
C14	0.018 (2)	0.025 (2)	0.023 (2)	−0.0074 (18)	−0.0032 (17)	−0.0011 (18)
C15	0.030 (3)	0.028 (2)	0.028 (2)	−0.016 (2)	−0.006 (2)	0.006 (2)
C16	0.027 (3)	0.036 (3)	0.034 (3)	−0.021 (2)	−0.006 (2)	−0.001 (2)
C17	0.023 (2)	0.033 (3)	0.025 (2)	−0.012 (2)	−0.0072 (19)	−0.0018 (19)
C18	0.015 (2)	0.020 (2)	0.019 (2)	−0.0037 (17)	−0.0023 (16)	−0.0050 (17)
C19	0.027 (2)	0.025 (2)	0.031 (3)	−0.011 (2)	−0.012 (2)	0.0074 (19)
C20	0.048 (3)	0.039 (3)	0.030 (3)	−0.017 (3)	−0.016 (2)	0.009 (2)
C21	0.033 (3)	0.042 (3)	0.038 (3)	−0.004 (2)	−0.014 (2)	0.005 (2)
C22	0.021 (2)	0.018 (2)	0.024 (2)	−0.0035 (17)	−0.0099 (18)	−0.0028 (17)
C23	0.021 (2)	0.028 (3)	0.043 (3)	−0.002 (2)	−0.016 (2)	0.001 (2)
C24	0.025 (2)	0.031 (3)	0.028 (2)	−0.007 (2)	−0.0051 (19)	0.003 (2)
C25	0.022 (2)	0.023 (2)	0.029 (2)	−0.0064 (19)	−0.0084 (19)	0.0012 (19)
C26	0.033 (3)	0.042 (3)	0.047 (3)	−0.014 (2)	−0.022 (2)	0.011 (2)
C27	0.017 (2)	0.026 (2)	0.026 (2)	−0.0059 (18)	−0.0043 (18)	0.0023 (19)
C28	0.072 (4)	0.037 (3)	0.028 (3)	−0.005 (3)	−0.007 (3)	−0.003 (2)
C29	0.020 (2)	0.043 (3)	0.020 (2)	−0.011 (2)	−0.0021 (18)	−0.003 (2)
C30	0.029 (3)	0.047 (3)	0.037 (3)	−0.020 (2)	−0.008 (2)	0.003 (2)
C31	0.015 (2)	0.033 (3)	0.037 (3)	−0.0052 (19)	−0.0066 (19)	−0.003 (2)
C32	0.077 (5)	0.040 (4)	0.057 (4)	0.001 (3)	−0.003 (4)	−0.017 (3)
C33	0.018 (2)	0.032 (3)	0.028 (2)	−0.0070 (19)	−0.0059 (18)	0.005 (2)
C34	0.036 (3)	0.038 (3)	0.033 (3)	−0.014 (2)	−0.018 (2)	0.011 (2)
C35	0.031 (3)	0.022 (2)	0.039 (3)	−0.008 (2)	−0.016 (2)	0.003 (2)
C36	0.048 (3)	0.032 (3)	0.053 (3)	−0.021 (3)	−0.026 (3)	0.017 (3)

### Geometric parameters (Å, º)

**Table:**

Zr1—N5	2.036 (4)	C18—C22	1.519 (6)
Zr1—N2	2.122 (4)	C19—C21	1.520 (7)
Zr1—N1	2.129 (4)	C19—C20	1.524 (7)
Zr1—N4	2.160 (4)	C19—H19	0.98
Zr1—N3	2.402 (4)	C20—H20A	0.96
N1—C1	1.417 (5)	C20—H20B	0.96
N1—H1A	0.890 (2)	C20—H20C	0.96
N2—C13	1.407 (5)	C21—H21A	0.96
N2—H2	0.87 (5)	C21—H21B	0.96
N3—C25	1.470 (6)	C21—H21C	0.96
N3—C27	1.473 (6)	C22—C24	1.522 (6)
N3—H3A	0.84 (5)	C22—C23	1.546 (6)
N4—C29	1.465 (6)	C22—H22	0.98
N4—C31	1.465 (6)	C23—H23A	0.96
N5—C33	1.469 (6)	C23—H23B	0.96
N5—C35	1.470 (6)	C23—H23C	0.96
C1—C6	1.417 (6)	C24—H24A	0.96
C1—C2	1.418 (6)	C24—H24B	0.96
C2—C3	1.398 (6)	C24—H24C	0.96
C2—C7	1.515 (6)	C25—C26	1.524 (6)
C3—C4	1.376 (7)	C25—H25A	0.97
C3—H3	0.93	C25—H25B	0.97
C4—C5	1.389 (7)	C26—H26A	0.96
C4—H4	0.93	C26—H26B	0.96
C5—C6	1.399 (6)	C26—H26C	0.96
C5—H5	0.93	C27—C28	1.511 (7)
C6—C10	1.512 (7)	C27—H27A	0.97
C7—C9	1.518 (6)	C27—H27B	0.97
C7—C8	1.546 (6)	C28—H28A	0.96
C7—H7	0.98	C28—H28B	0.96
C8—H8A	0.96	C28—H28C	0.96
C8—H8B	0.96	C29—C30	1.525 (7)
C8—H8C	0.96	C29—H29A	0.97
C9—H9A	0.96	C29—H29B	0.97
C9—H9B	0.96	C30—H30A	0.96
C9—H9C	0.96	C30—H30B	0.96
C10—C12	1.525 (7)	C30—H30C	0.96
C10—C11	1.537 (7)	C31—C32	1.508 (8)
C10—H10	0.98	C31—H31A	0.97
C11—H11A	0.96	C31—H31B	0.97
C11—H11B	0.96	C32—H32A	0.96
C11—H11C	0.96	C32—H32B	0.96
C12—H12A	0.96	C32—H32C	0.96
C12—H12B	0.96	C33—C34	1.531 (6)
C12—H12C	0.96	C33—H33A	0.97
C13—C14	1.413 (6)	C33—H33B	0.97
C13—C18	1.427 (6)	C34—H34A	0.96
C14—C15	1.396 (6)	C34—H34B	0.96
C14—C19	1.511 (6)	C34—H34C	0.96
C15—C16	1.375 (7)	C35—C36	1.528 (7)
C15—H15	0.93	C35—H35A	0.97
C16—C17	1.387 (7)	C35—H35B	0.97
C16—H16	0.93	C36—H36A	0.96
C17—C18	1.378 (6)	C36—H36B	0.96
C17—H17	0.93	C36—H36C	0.96
			
N5—Zr1—N2	115.34 (14)	C21—C19—H19	107
N5—Zr1—N1	116.93 (14)	C20—C19—H19	107
N2—Zr1—N1	126.85 (14)	C19—C20—H20A	109.5
N5—Zr1—N4	100.60 (14)	C19—C20—H20B	109.5
N2—Zr1—N4	90.93 (15)	H20A—C20—H20B	109.5
N1—Zr1—N4	88.60 (14)	C19—C20—H20C	109.5
N5—Zr1—N3	95.87 (13)	H20A—C20—H20C	109.5
N2—Zr1—N3	83.79 (14)	H20B—C20—H20C	109.5
N1—Zr1—N3	82.12 (14)	C19—C21—H21A	109.5
N4—Zr1—N3	163.41 (14)	C19—C21—H21B	109.5
C1—N1—Zr1	138.4 (3)	H21A—C21—H21B	109.5
C1—N1—H1A	105 (3)	C19—C21—H21C	109.5
Zr1—N1—H1A	111 (3)	H21A—C21—H21C	109.5
C13—N2—Zr1	133.5 (3)	H21B—C21—H21C	109.5
C13—N2—H2	109 (3)	C18—C22—C24	110.6 (4)
Zr1—N2—H2	111 (3)	C18—C22—C23	113.1 (4)
C25—N3—C27	114.4 (4)	C24—C22—C23	111.1 (4)
C25—N3—Zr1	117.5 (3)	C18—C22—H22	107.3
C27—N3—Zr1	120.5 (3)	C24—C22—H22	107.3
C25—N3—H3A	99 (4)	C23—C22—H22	107.3
C27—N3—H3A	106 (4)	C22—C23—H23A	109.5
Zr1—N3—H3A	93 (4)	C22—C23—H23B	109.5
C29—N4—C31	114.7 (4)	H23A—C23—H23B	109.5
C29—N4—Zr1	116.7 (3)	C22—C23—H23C	109.5
C31—N4—Zr1	125.7 (3)	H23A—C23—H23C	109.5
C33—N5—C35	114.2 (4)	H23B—C23—H23C	109.5
C33—N5—Zr1	124.4 (3)	C22—C24—H24A	109.5
C35—N5—Zr1	121.4 (3)	C22—C24—H24B	109.5
C6—C1—N1	120.8 (4)	H24A—C24—H24B	109.5
C6—C1—C2	119.6 (4)	C22—C24—H24C	109.5
N1—C1—C2	119.6 (4)	H24A—C24—H24C	109.5
C3—C2—C1	119.4 (4)	H24B—C24—H24C	109.5
C3—C2—C7	119.5 (4)	N3—C25—C26	114.5 (4)
C1—C2—C7	120.9 (4)	N3—C25—H25A	108.6
C4—C3—C2	121.0 (4)	C26—C25—H25A	108.6
C4—C3—H3	119.5	N3—C25—H25B	108.6
C2—C3—H3	119.5	C26—C25—H25B	108.6
C3—C4—C5	119.9 (4)	H25A—C25—H25B	107.6
C3—C4—H4	120.1	C25—C26—H26A	109.5
C5—C4—H4	120.1	C25—C26—H26B	109.5
C4—C5—C6	121.4 (5)	H26A—C26—H26B	109.5
C4—C5—H5	119.3	C25—C26—H26C	109.5
C6—C5—H5	119.3	H26A—C26—H26C	109.5
C5—C6—C1	118.7 (4)	H26B—C26—H26C	109.5
C5—C6—C10	121.0 (4)	N3—C27—C28	112.3 (4)
C1—C6—C10	120.4 (4)	N3—C27—H27A	109.1
C2—C7—C9	115.3 (4)	C28—C27—H27A	109.1
C2—C7—C8	109.3 (4)	N3—C27—H27B	109.1
C9—C7—C8	110.1 (4)	C28—C27—H27B	109.1
C2—C7—H7	107.3	H27A—C27—H27B	107.9
C9—C7—H7	107.3	C27—C28—H28A	109.5
C8—C7—H7	107.3	C27—C28—H28B	109.5
C7—C8—H8A	109.5	H28A—C28—H28B	109.5
C7—C8—H8B	109.5	C27—C28—H28C	109.5
H8A—C8—H8B	109.5	H28A—C28—H28C	109.5
C7—C8—H8C	109.5	H28B—C28—H28C	109.5
H8A—C8—H8C	109.5	N4—C29—C30	114.9 (4)
H8B—C8—H8C	109.5	N4—C29—H29A	108.5
C7—C9—H9A	109.5	C30—C29—H29A	108.5
C7—C9—H9B	109.5	N4—C29—H29B	108.5
H9A—C9—H9B	109.5	C30—C29—H29B	108.5
C7—C9—H9C	109.5	H29A—C29—H29B	107.5
H9A—C9—H9C	109.5	C29—C30—H30A	109.5
H9B—C9—H9C	109.5	C29—C30—H30B	109.5
C6—C10—C12	114.6 (4)	H30A—C30—H30B	109.5
C6—C10—C11	110.6 (4)	C29—C30—H30C	109.5
C12—C10—C11	109.1 (4)	H30A—C30—H30C	109.5
C6—C10—H10	107.4	H30B—C30—H30C	109.5
C12—C10—H10	107.4	N4—C31—C32	114.6 (5)
C11—C10—H10	107.4	N4—C31—H31A	108.6
C10—C11—H11A	109.5	C32—C31—H31A	108.6
C10—C11—H11B	109.5	N4—C31—H31B	108.6
H11A—C11—H11B	109.5	C32—C31—H31B	108.6
C10—C11—H11C	109.5	H31A—C31—H31B	107.6
H11A—C11—H11C	109.5	C31—C32—H32A	109.5
H11B—C11—H11C	109.5	C31—C32—H32B	109.5
C10—C12—H12A	109.5	H32A—C32—H32B	109.5
C10—C12—H12B	109.5	C31—C32—H32C	109.5
H12A—C12—H12B	109.5	H32A—C32—H32C	109.5
C10—C12—H12C	109.5	H32B—C32—H32C	109.5
H12A—C12—H12C	109.5	N5—C33—C34	114.7 (4)
H12B—C12—H12C	109.5	N5—C33—H33A	108.6
N2—C13—C14	121.1 (4)	C34—C33—H33A	108.6
N2—C13—C18	120.0 (4)	N5—C33—H33B	108.6
C14—C13—C18	118.8 (4)	C34—C33—H33B	108.6
C15—C14—C13	119.5 (4)	H33A—C33—H33B	107.6
C15—C14—C19	120.0 (4)	C33—C34—H34A	109.5
C13—C14—C19	120.5 (4)	C33—C34—H34B	109.5
C16—C15—C14	121.5 (4)	H34A—C34—H34B	109.5
C16—C15—H15	119.3	C33—C34—H34C	109.5
C14—C15—H15	119.3	H34A—C34—H34C	109.5
C15—C16—C17	118.9 (4)	H34B—C34—H34C	109.5
C15—C16—H16	120.5	N5—C35—C36	115.0 (4)
C17—C16—H16	120.5	N5—C35—H35A	108.5
C18—C17—C16	122.3 (4)	C36—C35—H35A	108.5
C18—C17—H17	118.8	N5—C35—H35B	108.5
C16—C17—H17	118.8	C36—C35—H35B	108.5
C17—C18—C13	118.9 (4)	H35A—C35—H35B	107.5
C17—C18—C22	120.8 (4)	C35—C36—H36A	109.5
C13—C18—C22	120.2 (4)	C35—C36—H36B	109.5
C14—C19—C21	111.3 (4)	H36A—C36—H36B	109.5
C14—C19—C20	114.2 (4)	C35—C36—H36C	109.5
C21—C19—C20	109.9 (4)	H36A—C36—H36C	109.5
C14—C19—H19	107	H36B—C36—H36C	109.5
			
N5—Zr1—N1—C1	−96.8 (5)	N1—C1—C6—C10	−3.1 (6)
N2—Zr1—N1—C1	71.9 (5)	C2—C1—C6—C10	178.9 (4)
N4—Zr1—N1—C1	162.0 (5)	C3—C2—C7—C9	28.7 (6)
N3—Zr1—N1—C1	−4.2 (4)	C1—C2—C7—C9	−155.7 (4)
N5—Zr1—N2—C13	−105.4 (4)	C3—C2—C7—C8	−95.9 (5)
N1—Zr1—N2—C13	85.7 (4)	C1—C2—C7—C8	79.7 (5)
N4—Zr1—N2—C13	−3.2 (4)	C5—C6—C10—C12	−25.1 (6)
N3—Zr1—N2—C13	161.0 (4)	C1—C6—C10—C12	155.9 (4)
N5—Zr1—N3—C25	−167.4 (3)	C5—C6—C10—C11	98.6 (5)
N2—Zr1—N3—C25	−52.5 (3)	C1—C6—C10—C11	−80.3 (5)
N1—Zr1—N3—C25	76.1 (3)	Zr1—N2—C13—C14	−106.6 (4)
N4—Zr1—N3—C25	19.6 (7)	Zr1—N2—C13—C18	72.2 (5)
N5—Zr1—N3—C27	−19.4 (3)	N2—C13—C14—C15	−179.9 (4)
N2—Zr1—N3—C27	95.5 (3)	C18—C13—C14—C15	1.3 (6)
N1—Zr1—N3—C27	−135.9 (3)	N2—C13—C14—C19	−0.7 (6)
N4—Zr1—N3—C27	167.5 (4)	C18—C13—C14—C19	−179.5 (4)
N5—Zr1—N4—C29	−174.3 (3)	C13—C14—C15—C16	−1.3 (7)
N2—Zr1—N4—C29	69.7 (3)	C19—C14—C15—C16	179.5 (4)
N1—Zr1—N4—C29	−57.2 (3)	C14—C15—C16—C17	0.9 (7)
N3—Zr1—N4—C29	−1.4 (7)	C15—C16—C17—C18	−0.5 (7)
N5—Zr1—N4—C31	−14.6 (4)	C16—C17—C18—C13	0.5 (7)
N2—Zr1—N4—C31	−130.6 (4)	C16—C17—C18—C22	176.5 (4)
N1—Zr1—N4—C31	102.5 (4)	N2—C13—C18—C17	−179.8 (4)
N3—Zr1—N4—C31	158.3 (4)	C14—C13—C18—C17	−0.9 (6)
N2—Zr1—N5—C33	−14.4 (4)	N2—C13—C18—C22	4.2 (6)
N1—Zr1—N5—C33	155.6 (3)	C14—C13—C18—C22	−176.9 (4)
N4—Zr1—N5—C33	−110.5 (3)	C15—C14—C19—C21	90.7 (5)
N3—Zr1—N5—C33	71.5 (3)	C13—C14—C19—C21	−88.5 (5)
N2—Zr1—N5—C35	164.6 (3)	C15—C14—C19—C20	−34.4 (6)
N1—Zr1—N5—C35	−25.4 (4)	C13—C14—C19—C20	146.4 (4)
N4—Zr1—N5—C35	68.4 (3)	C17—C18—C22—C24	−90.1 (5)
N3—Zr1—N5—C35	−109.5 (3)	C13—C18—C22—C24	85.9 (5)
Zr1—N1—C1—C6	−105.2 (5)	C17—C18—C22—C23	35.2 (6)
Zr1—N1—C1—C2	72.9 (6)	C13—C18—C22—C23	−148.8 (4)
C6—C1—C2—C3	0.2 (6)	C27—N3—C25—C26	59.7 (5)
N1—C1—C2—C3	−177.8 (4)	Zr1—N3—C25—C26	−150.5 (3)
C6—C1—C2—C7	−175.4 (4)	C25—N3—C27—C28	63.2 (5)
N1—C1—C2—C7	6.6 (6)	Zr1—N3—C27—C28	−85.7 (5)
C1—C2—C3—C4	0.2 (7)	C31—N4—C29—C30	58.1 (6)
C7—C2—C3—C4	175.9 (4)	Zr1—N4—C29—C30	−140.0 (4)
C2—C3—C4—C5	−0.7 (7)	C29—N4—C31—C32	62.7 (6)
C3—C4—C5—C6	0.8 (7)	Zr1—N4—C31—C32	−97.4 (5)
C4—C5—C6—C1	−0.4 (7)	C35—N5—C33—C34	−56.0 (5)
C4—C5—C6—C10	−179.4 (4)	Zr1—N5—C33—C34	123.1 (4)
N1—C1—C6—C5	177.9 (4)	C33—N5—C35—C36	−54.4 (5)
C2—C1—C6—C5	−0.1 (6)	Zr1—N5—C35—C36	126.5 (4)

### Hydrogen-bond geometry (Å, º)

**Table:**

*D*—H···*A*	*D*—H	H···*A*	*D*···*A*	*D*—H···*A*
N3—H3*A*···N1	0.84 (5)	2.56 (5)	2.983 (5)	112 (4)
